# Underwater traction-assisted endoscopic submucosal dissection of a neuroendocrine tumor in the duodenal bulb

**DOI:** 10.1055/a-2695-4001

**Published:** 2025-09-11

**Authors:** Paolo Cecinato, Angelo Bruni, Liboria Laterza, Michele Dota, Nicola De Angelis, Rocco Maurizio Zagari, Giovanni Barbara

**Affiliations:** 1 18508 Gastroenterology Unit, IRCCS Azienda Ospedaliero-Universitaria di Bologna Policlinico di SantʼOrsola, Bologna, Italy; 2 9296 Department of Medical and Surgical Sciences, University of Bologna, Bologna, Italy; 3 19001 Department of Internal Medicine and Medical Therapy, University of Pavia, Pavia, Italy; 4 18560 Unit of Robotic and Minimally Invasive Digestive Surgery, Department of Surgery, University Hospital Arcispedale SantʼAnna of Ferrara, Cona, Italy; 5 9299 Department of Translational Medicine and LTTA Centre, University of Ferrara, Ferrara, Italy; 6 18508 Esophagus and Stomach Organic Diseases Unit, IRCCS Azienda Ospedaliero-Universitaria di Bologna Policlinico di SantʼOrsola, Bologna, Italy

A 68-year-old man with a 15-mm subepithelial lesion (SEL) on the anteroinferior wall of the duodenal bulb, just distal to the pylorus ( Fig. 1 **a** ) was referred for further management. An endoscopic ultrasound (EUS) was performed, which identified a 15-mm oval-shaped, hypoechoic, submucosal lesion, with a finely inhomogeneous echostructure and peripheral vascular signals. Fine-needle biopsy (FNB) with a 22G Trident needle (Micro-Tech, Nanjing, China) confirmed the diagnosis of a neuroendocrine tumor (NET).

**Fig. 1:**
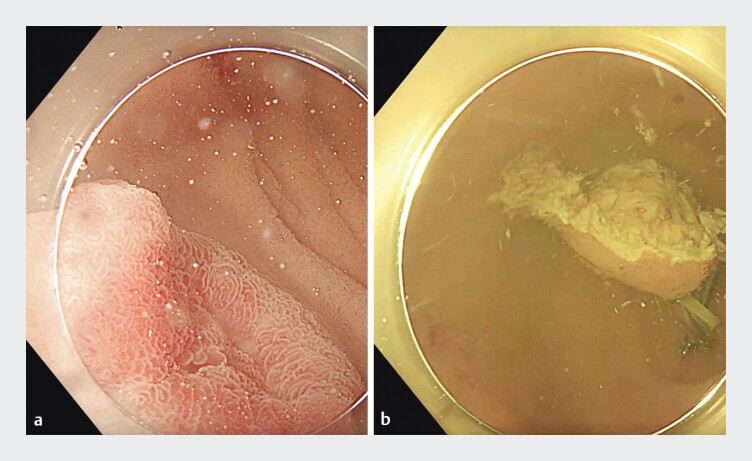
Endoscopic images showing: **a** a neuroendocrine tumor (NET) located on the anteroinferior wall of the duodenal bulb; **b** the resected specimen of the duodenal bulb NET following underwater traction-assisted endoscopic submucosal dissection.

Duodenal NETs are rare neoplasms, for which guidelines recommend resection when the lesion is ≤20 mm in size and no lymph node involvement is detected 1 2 . The role of endoscopic resection remains debatable owing to the potential risk of incomplete removal 3 . Endoscopic submucosal dissection (ESD) offers the potential to achieve en bloc resection with clear margins, making it an attractive option in selected cases 4 .

An underwater ESD was therefore performed in this patient. After the duodenal bulb had been filled with saline solution, the lesion was resected en bloc using a 2-mm T-type Gold-knife (Micro-Tech), with the rubber band and clip traction technique applied on the contralateral side of the bulb ( Fig. 1 **b** ). Careful dissection with prophylactic coagulation of prominent vessels using the Gold-knife was performed to preserve the muscular layer and ensure procedural safety ( Fig. 2 ; Video 1 ).

**Fig. 2:**
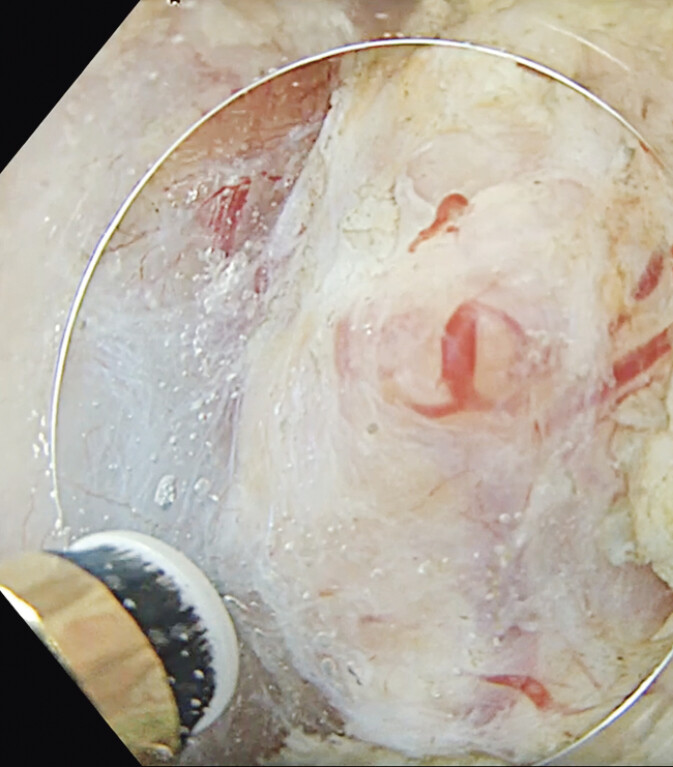
Endoscopic image of the exposed muscular layer during resection of the neuroendocrine tumor, demonstrating precise submucosal dissection.

Underwater traction-assisted endoscopic submucosal dissection of a duodenal neuroendocrine tumor located in a challenging anatomical site.Video 1

Histopathological analysis confirmed an R1 resection of a well-differentiated G1 NET, with infiltration of the mucosal and submucosal layers up to a depth of 6 mm. Biopsies were performed at the 6-month follow-up endoscopy, even though there was no visible local recurrence, and these confirmed no evidence of residual or recurrent disease ( Fig. 3 ).

**Fig. 3:**
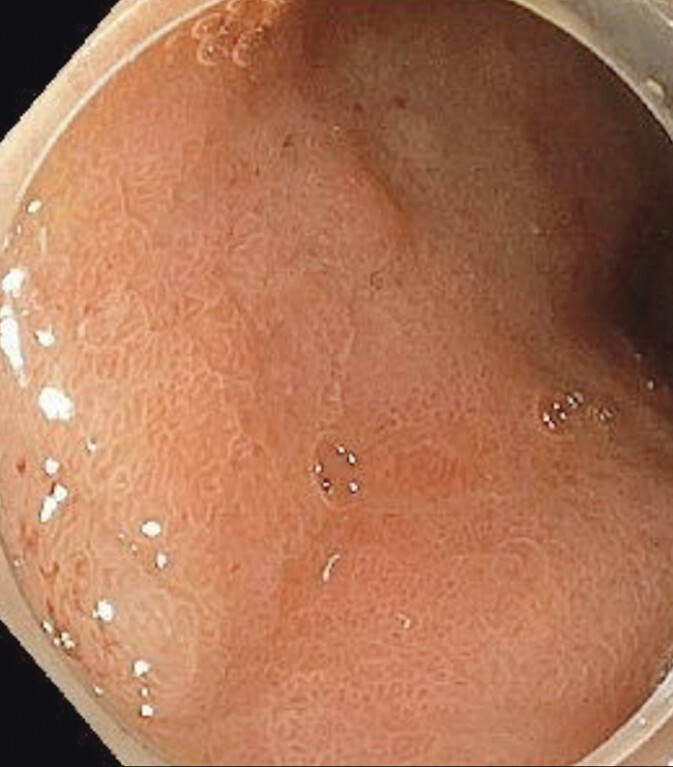
Endoscopic image during follow-up at 6 months showing complete healing of the resection site with no evidence of local recurrence.

This case demonstrates that combining traction techniques that have been previously shown to be effective 5 with underwater ESD significantly enhances the visualization and differentiation of the gastrointestinal wall layers. This approach facilitates precise dissection, enables complete tumor resection, and preserves the integrity of the muscular layer. Furthermore, underwater traction-assisted dissection improves confidence in achieving curative resections, even when histological analysis indicates R1 or undefined margins. Such findings can often be attributed to thermal artifacts from the dissection device, which may compromise optimal histopathological assessment.

Endoscopy_UCTN_Code_TTT_1AO_2AG_3AD

## Notes

Endoscopy E-Videos https://eref.thieme.de/e-videos
*E-Videos* is an open access online section of the journal *Endoscopy* , reporting on interesting cases and new techniques in gastroenterological endoscopy. All papers include a high-quality video and are published with a Creative Commons CC-BY license. Endoscopy E-Videos qualify for HINARI discounts and waivers and eligibility is automatically checked during the submission process. We grant 100% waivers to articles whose corresponding authors are based in Group A countries and 50% waivers to those who are based in Group B countries as classified by Research4Life (see: https://www.research4life.org/access/eligibility/ ). This section has its own submission website at https://mc.manuscriptcentral.com/e-videos .
